# Current Understanding of the Pathogenesis of ANCA-Associated Vasculitis and Novel Treatment Options Targeting Complement Activation

**DOI:** 10.3390/life15050756

**Published:** 2025-05-08

**Authors:** Konstantinos Drouzas, Petros Kalogeropoulos, George Liapis, Sophia Lionaki

**Affiliations:** 1 Department of Nephrology, 2nd Propaedeutic Internal Medicine, Attikon University Hospital, Medical School, National and Kapodistrian University of Athens, 12461 Athens, Greece; petroskalogeropoulos@hotmail.com; 2 1st Department of Pathology Medical School, National and Kapodistrian University of Athens and Laikon Hospital, 11527 Athens, Greece; gliapis@gmail.com

**Keywords:** ANCA glomerulonephritis (ANCA-GN), granulomatosis with polyangiitis (GPA), microscopic polyangiitis (MPA), complement activation, B-depletion, avacopan

## Abstract

Anti-neutrophil cytoplasmic antibody (ANCA)-associated vasculitis (ANCA-vasculitis) is an autoimmune disease characterized by inflammation and necrosis of small or medium vessels. In the past, the role of the complement in the pathogenesis of ANCA-vasculitis has been underestimated, due to the paucity of the complement at sites of injured glomeruli. Following evidence from animal models of the major role of the complement in pathogenesis of ANCA-vasculitis, the complement has again attracted interest. Immunohistology analysis of pauci-immune glomerulonephritis—ANCA glomerulonephritis (ANCA-GN)—reveals the presence of complement products and membrane attack complex, suggesting their involvement in the disease process. Researchers emphasize the complement classical or lectin pathway as a contributor to the development of ANCA-vasculitis. The era of targeted therapies to suspend the complement activation as a therapy for ANCA-vasculitis has arrived, and thus, the comprehension of its role is very important. This review summarizes recent insights on the important role of complement activation in the development of ANCA-vasculitis as well as the emerging therapeutic possibilities that target complement components for the treatment of this condition.

## 1. Introduction

Anti-neutrophil cytoplasmic antibody (ANCA)-associated vasculitides (ANCA-vasculitis) is a disease that affects small-sized vessels and by definition medium-sized arteries, which may lead to multisystem organ involvement. The clinical phenotypes of these diseases include microscopic polyangiitis (MPA), granulomatosis with polyangiitis (GPA), eosinophilic granulomatosis with polyangiitis (EGPA), and renal-limited vasculitis [1,2]. The prevalence of MPA ranges from 9 to 94 cases per million persons, and that of GPA, 2.3 to 146 cases per million persons [3]. Caucasian populations have been found to have twofold higher incidence for these diseases, compared with other ethnicities, while both GPA and MPA most commonly occur in older adults, affecting both genders equally [3,4,5,6]. These diseases can affect any organ, with the upper and lower respiratory tract and the kidneys being the most commonly involved [3,7]. Kidney involvement is frequent [8,9], and the clinical presentation is that of a rapidly progressive glomerulonephritis (RPGN), where patients experience a kidney failure over days or weeks typically associated with microscopic hematuria with dysmorphic red blood cells and proteinuria [8]. The typical histologic feature is that of pauci-immune necrotizing and crescentic glomerulonephritis, i.e., with vasculitis with infiltrating neutrophils and monocytes and meager deposition of reactants such as the complement [2,9,10]. Historically, the role of the complement has been considered secondary in ANCA-vasculitis. Initially, complement activation was not suspected as a player in the pathogenesis because of the relatively scanty complement deposition at the affected sites. In contrast, extensive localization of complement components was identified at sites of inflammation induced by recognized forms of immune-complex-mediated inflammation, which could involve extensive complement activation [11].

## 2. ANCA-Vasculitis Pathogenesis

Anti-neutrophil cytoplasmic autoantibodies are autoantibodies that firstly target antigens in the cytoplasm of neutrophils and secondly target lysosomes of monocytes that are peroxidase-positive. Davies et al. was the first to describe them [12]. It was then revealed that the majority of patients that suffer from pauci-immune crescentic glomerulonephritis are found to have circulated ANCA, independently of the presence of systemic vasculitis or not [13]. A strong relationship between ANCA positivity and ANCA-vasculitis diagnosis has been established, and thus, ANCA testing plays an important role in the classification and diagnosis of this group of diseases [13,14,15,16,17]. Two types of ANCA assays are used. The first one is indirect immunofluorescence assay which utilizes alcohol-fixed buffy coat leukocytes, and the second one is enzyme-linked immunosorbent assay (ELISA) which uses specific antigens. The methodology of ELISA is used for detection of antibodies against myeloperoxidase (MPO) and proteinase 3 (PR3) antigens, known as and MPO-ANCA and PR3-ANCA. Ninety percent of perinuclear ANCA are MPO-ANCA, and nearly 90% of cytoplasmic ANCA are PR3-ANCA. They are both located in the cytosol, and they are inaccessible to antibody binding. Neutrophil exposure to pro-inflammatory substances such as tumor necrosis factor and inteleukin-1β is a prerequisite to induce ANCA autoantigen translocation to the cell surface (“priming”) [18]. ANCA binding to neutrophils promotes neutrophil–endothelial cell interactions and enhances the production of reactive oxygen species, lytic enzymes, and neutrophil extracellular traps (NETs), leading to microvascular injury [19,20,21]. These are webs of decondensed chromatin and a key player in the host’s innate immune response, binding and killing pathogens [21], which in ANCA-vasculitis have been proposed as potential triggers for ANCA immunogenicity [22], leading to activation of the alternative complement cascade and damage of the endothelial cells locally [23,24]. Activated neutrophils secrete pro-inflammatory factors and cytokines such as BAFF (B-cell activating factor) which activates B-cells [25,26]. B-cells also have an essential role in the pathogenesis of ANCA-vasculitis, which is confirmed by clinical trials with anti-CD20 therapies [27,28]. With transient elimination of the B-cells, the production of ANCAs is reduced, and their contribution to antigen presentation and cytokine production is limited. Despite the fact that ANCA-vasculitis is an antibody-related disease, the role of T-cells is equally important in its pathogenesis. The population of T regulatory cells, which regulate the expression and progression of autoimmunity by suppressing autoreactive T-cells, appears to be reduced in ANCA-vasculitis [18,29,30]. Th1 T-cells are also activated, which produce IFNγ and TNFα, inducing macrophage migration, as well as increased Th17 T-cell activity, which in turn contributes to neutrophil recruitment. The migration of all these cells leads to the formation of granulomas in GPA [31].

In addition, the BLyS family of ligands and receptors has been implicated in the pathogenesis of ANCA-vasculitis, including mainly the ligands APRIL and BLyS. The most important activities of BLyS are the ones related to the development of primary B-cells. Serum concentrations of BAFF, APRIL, and other cytokines, including IL-4, IL-6, IL-10, and IL-13, can be implicated in the differentiation of B-cell lineages [32], while significant increases in BAFF, APRIL, IL-4, and IL-6 were observed in patients with ANCA-vasculitis [33].

It has been postulated that upregulation of BAFF and APRIL may be induced to promote the repopulation of circulating B-cells, a process that may be dependent on residual pathological B-cells in the tissues [34]. Accordingly, increases in serum levels of BAFF and APRIL may be used as general indicators for monitoring remission in autoimmune diseases, including ANCA-vasculitis, in which B-cell lineage is implicated in their pathogenesis. Conversely, the activation of immunocompetent cells, which persistently produce BAFF and APRIL, can participate in ANCA-vasculitis relapse. Patients with ANCA-vasculitis also demonstrated significantly higher concentrations of IL-4 than Henoch–Schönlein in this study, although no differences were found in the concentrations of IL-4, IL-10, or IL-13 in patients with ANCA-vasculitis [35]. A characteristic of BAFF/APRIL receptors in ANCA-vasculitis is that the higher expression of the transmembrane activator and calcium modulator and cyclophilin ligand interactor on CD19+ cells, immature B-cells, and PB/PC remained even in the remission state of these patients, implying persistent aberrant signaling of BAFF/APRIL may contribute to disease relapse [36].

More recently, a comprehensive analysis using spectral flow cytometry provided novel insights into the expression patterns of key inhibitory and stimulatory immune molecules on B-cells in GPA, suggesting that changes in the expression of molecules such as FcγRIIB, CD21, CD86, and CD22 on specific B-cell populations, combined with the strong association of FcγRIIB, BTLA, and CD21 expression with disease activity, reveal the complexity of immune regulation in the pathogenesis of GPA [37].

Participation of ANCAs themselves in disease pathogenesis is suggested by many experimental and clinical studies and by the efficacy of plasma exchange and anti-B-cell therapy. The study by Xiao et al. showed that mice developed pauci-immune vasculitis when given anti-MPO immunoglobulin intravenously [38]. Likewise, the immunization of rats with human MPO resulted in the development of anti-MPO antibodies which are directed against rat leucocytes and cause extensive microvascular injury [19]. The first evidence in humans was seen in a case where anti-MPO antibodies were transferred from the mother to the neonate during delivery, resulting in the development of pulmonary–renal syndrome [39]. Yet, a pathogenic role of ANCA has also been established by numerous in vitro studies, where both MPO-ANCA and PR3-ANCA activate neutrophils to release inflammatory mediators [40,41,42]. Activated neutrophils can kill cultured endothelial cells or cause integrin and cytokine receptor-mediated adherence to cultured endothelial cells and transmigration across the endothelial layer [43]. The interaction between ANCA-activated neutrophils and vessels causes unregulated adhesion of molecules in glomerular lesions of human kidney specimens [44]. Evidence that ANCAs are pathogenic comes from in vivo studies in which passive transfer of anti-MPO-ANCA IgG or anti-MPO lymphocytes into either immune-competent mice or Rag2(-/-) mice led to induction of pauci-immune crescentic glomerulonephritis [38]. All mice developed glomerular lesions remarkably similar to human disease within 6 days from injection, while some developed necrotizing arteritis, pulmonary capillaritis, leukocytoclastic angiitis, and necrotizing granulomatosis inflammation [38,45,46,47]. Likewise, the immunization of rats with human MPO, which developed anti-MPO antibodies that cross-react with rat and human MPO, caused them focal segmental pauci-immune glomerulonephritis and focal pulmonary capillaritis [19].

The clinical syndromes of pauci-immune vasculitis are associated with the type of ANCA antibodies involved. To elaborate, patients with renal-limited disease or patients with any form of vasculitis but without any proof of granulomas are more likely to have MPO-ANCA. On the contrary, patients with evidence for necrotizing granulomatous inflammation are most likely to have PR3-ANCA. The above was shown in a clinical study with 523 patients with vasculitis, where 81% of those with kidney-limited disease were positive for MPO-ANCA, while 94% of patients with bone destruction or saddle nose deformity were positive for PR3-ANCA [48]. Moreover, in this study, 79% of patients with histological proof of granulomatous inflammation had PR3-ANCA compared to the 21% of them who had MPO-ANCA. Therefore, there is a clear clinical distinction between diseases associated with MPO-ANCA and PR3-ANCA, an observation which is essential with respect to the classification of the disease based on antibody specificity [48]. At the same time, a study from Lyon et al. tried to show the genetic component of the disease, associated with ANCA specificity, i.e., the response against PR3 autoantigen is a central pathogenic feature of PR3 ANCA-vasculitis [49]. However, there are patients with typical clinical or histopathological vasculitic syndrome who are repeatedly found to be negative for ANCA antibodies. In this regard, a novel assay was created to identify the specific target epitopes for ANCA [44], which led to the discovery of MPO-ANCA in patients with ANCA-negative disease reacting against a sole linear sequence. Autoantibodies against this epitope had pathogenic properties, as demonstrated in vitro. The presence of a fragment of ceruloplasmin in serum was shown to be a confounder for its serological detection, indicating that different epitopes may play a role in autoantibody diversity in this and other autoimmune diseases [50].

## 3. Risk Factors and Potential Initiating Events

Several factors contribute to the pathogenesis of ANCA-vasculitis, such as environmental and genetic factors, various infections, innate and adaptive immune system characteristics, and the intensity and duration of the injury [51]. In this regard, a correlation between PR3-ANCA serotype and certain genes encoding alpha-1 antitrypsin, PR3, and HLA-DP has been reported, where anti-MPO-ANCA is associated with HLA-DQ [49,52,53]. Major histocompatibility complex(MHC) class II allele HLA-DRB1*15 is associated with the occurrence of PR3-ANCA-vasculitis among patients of African descent [5] and other genetic variants among Scandinavians or among Chinese patients with MPA [54,55,56]. Also, HLA-DPB1*0401 allele appears to increase the risk of recurrence in PR3-ANCA serotype [57]. Polymorphism rs62132293 of PRTN3 variant, encoding PR3, is associated with increased risk of a relapse in PR3-ANCA compared to MPO-ANCA disease, while a lack of HLA and autoantigen interaction observed during long-term remission signals immunologic non-responsiveness [58]. Epigenetic factors have an important role in the expression of genes encoding ANCAs as well in the activation of neutrophils [59,60,61,62,63]. Infectious factors like staphylococcus aureus [64,65] as well as several medications are connected with the pathogenesis of ANCA-vasculitis [66]. Among others, hydralazine, an antihypertensive agent [67,68], and propylthiouracil, which is used in patients with hyperthyroidism, have been associated with ANCA seropositivity [69,70] by accumulating in neutrophils, binding to alter the MPO antigen [71,72]. Epidemiological studies show clear correlation of the occurrence of ANCA-vasculitis and the exposure to silica [73,74,75] and smoking, with the risk being dose-dependent [76] in both cases.

## 4. Investigating the Role of Complement System in ANCA-Vasculitis

The complement is a tightly regulated network of proteins that is an important part of the host defense system, both innate and adaptive immunity. It also acts as an injury mediator in numerous autoimmune and auto-inflammatory conditions. The complement system is divided into three major cascades: classical (immune-complex-mediated), lectin (recognition of carbohydrate pattern on microbial and other surfaces), and alternative pathways. Each pathway is activated under different conditions, but all three pathways lead to a common pathway, where large amounts of C3 are deposited in the target cell, evoking lysis by the membrane attack complex (MAC) [77,78]. The complement system was initially thought not to play a major role in the pathogenesis of ANCA-vasculitis. This hypothesis relied on the absence of significant immunofluorescence complement staining in biopsy specimens, while most patients did not have normal C3 and C4 titers [79]. Emerging research on ANCA-vasculitis over the past few decades sheds light on the crucial role of the complement system in driving inflammation and tissue damage. Immunohistology analysis of pauci-immune glomerulonephritis reveals the presence of complement products and membrane attack complex, suggesting their involvement in the disease process [80,81]. Studies by Wu et al. and Xing et al. demonstrate the rise in the levels of C3a, C5a, soluble C5b-9 in patients with ANCA-vasculitis, compared to healthy individuals, further highlighting the complement’s contribution. Additionally, these studies showed lower levels of activation products during disease remission and in patients achieving long-term remission, implying a potential link between complement activity and disease state [80,82]. Furthermore, there are retrospective studies that correlate patients with pauci-immune glomerulonephritis and low serum complement C3 levels with a higher index of histopathological activity, including signs of thrombotic microangiopathy and high probability of treatment resistance [83,84,85,86,87]. Focusing on the alternative pathway, Xiao et al. employed knockout mice to show that C5 deficiency (necessary for all complement pathways) and C4 deficiency (classical and lectin pathways) prevented pauci-immune glomerulonephritis development, while factor B deficiency (alternative pathway) did not. This suggests the alternative pathway plays a more prominent role than the classical or lectin pathways. Afterwards, their research revealed that when primed neutrophils were exposed to anti-MPO and anti-PR3 IgG, there was a substantial increase in C3a production compared to the control group. This finding strongly implies that the activation of neutrophils which are induced from ANCA antibodies results in the release of substances that trigger the complement cascade [88]. Chen et al. explored the role of factor H, a crucial regulator of the alternative pathway, finding that it can prevent ANCA-induced neutrophil activation and endothelial damage. However, factor H from ANCA-vasculitis patients was less effective, suggesting potential abnormalities in its function [89]. Additionally, reactive oxygen species and proteolytic enzymes released during neutrophil activation might damage endothelial cells as occurs in patients with complement-related thrombotic microangiopathy, leading to the loss of alternative pathway regulators and further contributing to uncontrolled complement activation [90]. Complement factor H, when bound to neutrophils, can prevent ANCA-induced neutrophil activation. However, in patients with active ANCA-vasculitis, FH shows a reduced capacity to inhibit this activation. Myeloperoxidase, which is released from activated neutrophils by ANCA, binds to FH and hinders its regulatory activity on the complement system. This suggests that a deficiency in FH could intensify the feedback loop between neutrophil activation and the alternative complement pathway, thereby playing a role in the progression of ANCA-vasculitis [89,91]. Recently, analysis for common gene variants in the CFH/CFHR1-CFHR5 gene cluster detected CFB, C3, and CD46 (MCP) genes, along with determination of plasma levels for C3, C4, FH, FHR-1/2/5, FB properdin, in 102 individuals with ANCA-vasculitis (54 active and the remaining in remission off immunosuppression). Using a validation cohort of 100 individuals with pauci-immune glomerulonephritis, it was demonstrated that a common intragenic variant of the CFB gene affecting arginine at position 32 of the protein was associated with disease susceptibility. Deletion of CFHR3/CFHR1 genes as well as two CFH gene haplotypes (H1 and H2) was associated with disease severity. Interestingly, while a risk allele/haplotype may not be causative by itself but rather could be genetically linked to other causative variants within the same haplotype, the authors did not proceed with sequencing of the respective genes. A worse outcome was associated with alternative complement pathway activation. High basal FHR-1 levels or FH/FHR-1 ratios were shown to be determinants of more severe forms of ANCA glomerulonephritis. It however remains to be shown whether haplotypes associated with disease susceptibility or severity are by themselves causative, or—as is very often the case—are genetically linked to other causative variants within the same haplotype. Further, variations in FHR-1 or FH1 plasma levels might be mediated by yet unknown genetic determinants [92]. The complement anaphylatoxin C5a has a significant role in the pathogenesis of ANCA-vasculitis. Neutrophils activated by ANCA generate C5a, which then binds to the C5a receptor (C5aR) on neutrophils, which leads neutrophils to their priming. C5a promotes inflammation by various mechanisms. It increases vascular permeability, promotes the expression of endothelial cell adhesion molecules, and can attract more neutrophils [93,94]. Blocking the C5aR has been shown to prevent or reduce ANCA-induced glomerulonephritis in experimental models, and an inhibitor of human C5aR has been found to reduce glomerulonephritis [95,96].

## 5. Histopathological Evidence for the Role of Alternative Complement Pathway in Pathogenesis of ANCA-Vasculitis

**Figure 1:**
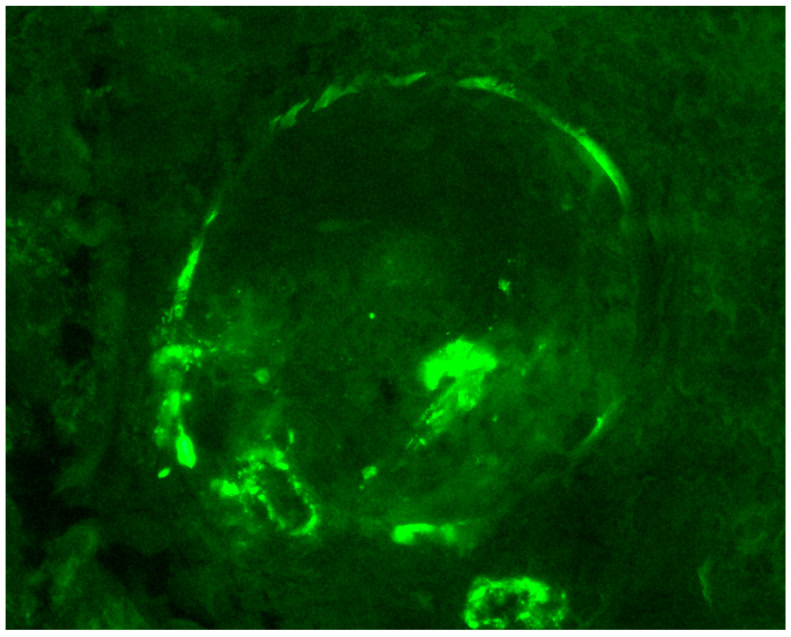
C3 is highlighted in the arteriole and Bowman capsule, while “trapping” in a small area of the glomerulus is seen, probably corresponding to an area of segmental glomerular necrosis; the rest of the glomerulus is negative (immunofluorescence, C3 ×400).

**Figure 2:**
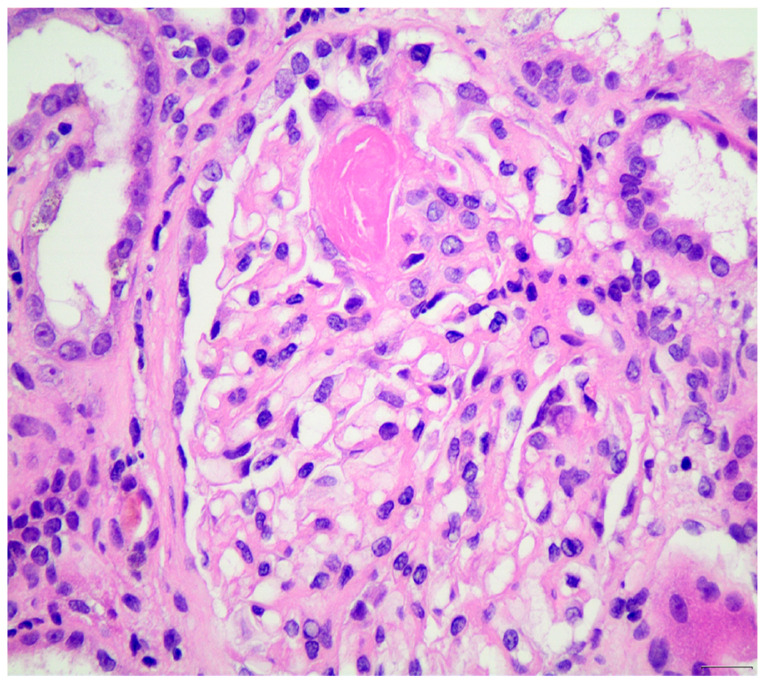
Fibrinoid necrosis in a glomerulus in the upper half of the image, from a case of ANCA-vasculitis (H&E ×400).

The role of the complement in the pathogenesis of ANCA-vasculitis had been underestimated in the past, due to the paucity of complement component at the sites of glomerular injury, as opposed to immune-complex-mediated vasculitis. Histopathology examination of the tissue samples in immunofluorescence usually does not reveal substantial complement deposition, except of “trapping” or traces, in areas of glomerular necrosis or sclerosis. This “trapping” is thought to be non-specific (Figure 1 and Figure 2). Furthermore, hypocomplementemia in ANCA-vasculitis is not encountered very often and only seen in a small portion of the cases; thus, evidence of systemic complement involvement in the pathogenesis is not clear. However, animal models have provided new data for the role of the complement in the pathogenesis of ANCA-vasculitis, therefore attracting research interest. Indeed, immunohistology showed that one third of the patients had focal complement deposition located at injury sites [11]. Thus, it was supposed that alternative complement activation participates in the pathogenesis of ANCA-vasculitis, even if lesions do not have robust complement deposition by immunohistology [97]. Recent studies showed that C3d, C4d, and C5b-9 staining was detected in the majority of renal biopsies, mostly in a patchy pattern, especially for C5b-9, while C3d and properdin staining was associated with cellular crescent formation in a study [98]. Authors hypothesized that local immune complexes are quickly degraded in ANCA-vasculitis and this fast complement disintegration explains why deposits are not detected by electron microscopy examination. Focal and segmental glomerular staining for C3c was also detected in another study, especially in areas of inflammation and necrosis [99]. Moreover, we previously showed that hypocomplementemia at diagnosis is associated with higher histopathological activity and high probability of treatment resistance [71]. Glomerular deposition of C3 in MPO-ANCA-vasculitis has been associated with worse renal outcomes [100].

Other studies showed that complement C4d deposition in venules is correlated with serum uric acid levels [101], while another study showed that C3 deposits are mostly localized to the glomerular tuft overlapping with peritubular capillaries. The latter study supported the concept of intrarenal C3 synthesis, given the fact that intrarenal complement C3 deposits were not combined with consumption of serum C3 levels [102].

It is well known from experiments that isolation of IgG from patients with MPO- ANCA or PR3-ANCA and incubation with human neutrophils can release factors that activate the complement. On the contrary, IgG from healthy individuals cannot generate this phenomenon [88]. ANCA-activated neutrophils can activate the complement by generating C3a and C5a, which, in turn, activate more neutrophils. This finding is in agreement with other studies that showed activated neutrophils can activate the complement. In particular, activation of C5 is important for inducing ANCA-vasculitis in animal models. Inhibition of C5, by using a C5-inhibiting monoclonal antibody (BB5.1), prevents ANCA-vasculitis and reduces glomerular inflammation [103]. These data suggest that neutrophils after ANCA activation activate the complement, especially the alternative pathway, which recruits more neutrophils for ANCA activation, continuing an amplification loop. In addition, C5a acts in two ways, by recruiting additional neutrophils through chemotaxis and also priming neutrophils for ANCA activation [93]. Activated neutrophils release properdin, promoting the alternative pathway and generating the anaphylatoxin C5a, which binds to C5a receptors on neutrophils, resulting in further neutrophil priming and activation. Moreover, activated neutrophils may be subjected to a form of cell death (NETosis), where neutrophil extracellular traps (NETs) that contain entrapped MPO, PR3, and complement components as a web are extruded from the cell [104].

Immunohistological studies showed that C3d, Factor B, and Factor P can be deposited in glomeruli and small vessels, in sites where there is active inflammation, suggesting a role for alternative complement activation in ANCA-vasculitis. On the contrary, Mannose-binding lectin and C4d were not detected in ANCA-vasculitis patients, indicating that the Mannose-binding pathway and classical pathway are not involved in ANCA-vasculitis [80]. Fc receptor engagement is also important, since modulation of ANCA IgG glycosylation may reduce pathogenicity by eliminating Fc receptor-mediated activation of leukocytes and complement [105]. An alternative complement pathway can also be triggered by platelets that are activated by thrombin-PARs pathway [106]. Current studies suggest that genetic and plasma components of the alternative pathway can affect ANCA-vasculitis susceptibility and severity, indicating that Factor H-related proteins-1 can be a therapeutic target in ANCA-vasculitis [92].

However, although there is emerging and critical evidence regarding the role of the complement alternative pathway in ANCA-vasculitis, there are currently certain limitations and an absence of accurate guidelines for utilizing these findings for patient management. These limitations include the lack of a clear serum or tissue-deposited C3 cut-off correlating with outcome or mortality risk, the normal serum C3 levels in the majority of ANCA-vasculitis patients, the lack of a systemic validation, especially for studies correlating clinical and histological variables, and of course, the lack of association between serum C3 levels and C3 deposition at the sites of tissue damage. In addition, the similar kidney outcome irrespective of C3 deposition, as it is suggested in some studies [107], does not justify the use of complement fractions as specific biomarkers of ANCA-vasculitis outcomes, at least at present.

## 6. Current Therapeutic Targets for ANCA-Vasculitis and Glomerulonephritis

The goal of therapy in patients with pauci-immune glomerulonephritis and vasculitis is to achieve a rapid, long-standing remission. The initial phase primarily aims at achievement of remission through suppression of the inflammatory process, while maintenance therapy aims to maintain the status of remission and prevent potential disease reactivation, i.e., relapses. In recent years, initial regimens consist of glucocorticoids and cyclophosphamide or rituximab which are considered equivalent in terms of efficacy and frequency and severity of adverse events [108]. The choice between cyclophosphamide and rituximab depends on various factors, including the severity of the disease, availability of rituximab, and patient-specific concerns such as fertility, alopecia, and malignancy. However, for patients with organ- or life-threatening ANCA-vasculitis, i.e., rapidly progressive glomerulonephritis or pulmonary hemorrhage, the induction regimen unavoidably includes intravenous pulses of methyl-prednisolone combined with cyclophosphamide with plasma exchange or rituximab [109]. Although according to recent guidelines, plasma exchange is no longer a first-line therapy, it is used in cases with aggressive disease. The recommendation (108) is that plasma exchange should only be considered under certain conditions as the ones mentioned above. The evidence for this is based on the data from the PEXIVAS Trial, to date the largest of its kind, which failed to demonstrate that plasma exchange delayed the time to kidney failure or death for patients with ANCA-vasculitis presenting with GFR < 50 mL/min per 1.73 m^2^ or alveolar hemorrhage over a median follow-up of 2.9 years [110]. In addition to certain methodological problems associated with this study, others have shown that kidney biopsy findings may play a significant role in the decision, i.e., the presence of active inflammation without significant glomerulosclerosis identifies patients most likely to benefit from plasma exchange [111]. Yet, a meta-analysis showed a reduction in the risk of kidney failure after 12 months with plasma exchange, accompanied by an increase in the risk of serious infections [112]. In this meta-analysis, a randomized study of the European Vasculitis Group investigated the influence of plasma exchange in patients with serum creatinine >5.7 mg/dL, with the greatest influence on improved renal function after 12 months. However, the long-term data from this study no longer showed any benefit after a longer follow-up period [112]. Thus, the role of plasma exchange in ANCA-vasculitis still remains a controversial issue.

Importantly, however, the PEXIVAS trial showed that the reduction rate of glucocorticoids was non-inferior in the reduced-glucocorticoids-dose group compared to the standard-dose group, regarding the occurrence rate of either ESKD or mortality, with the condition that cyclophosphamide or rituximab was used in combination with glucocorticoids [110]. Accordingly, during the remission maintenance, it was advised that the glucocorticoids dose should be reduced and stopped [113].

## 7. Targets: B-Cells, T Lymphocytes, Inflammatory Agents

Recent insights into the causes of ANCA-vasculitis have clearly shown that ANCAs can induce the disease, while other factors, i.e., genetic, epigenetic, and environmental, probably play important roles in patients’ clinical course and can modify the disease phenotype and differentiate the related manifestations. Considering this, it was almost expected that therapies acting through B-lymphocyte depletion would result in decreases in ANCA titers with beneficial effect on ANCA-vasculitis. In addition, B-cell depletion therapy is believed to have a more general effect on the pathogenesis of autoimmunity by B-cell remodeling in the long term and potentially T-cell regulatory functions. Therefore, the fact that anti-B-cell therapies diminish the propensity for disease relapse in patients with ANCA-vasculitis comes as a consequence of all the related ANCAs’ properties, which have been shown to trigger and perpetuate the disease [39,114]. One option is to use an invasive methodology, i.e., plasma exchange to remove ANCAs from the circulation with all the adverse events coming with it, including the use of central catheter and sessions performed using the appropriate machine every other day. Since the introduction of rituximab in the therapeutic armory of autoimmune diseases, ANCAs can be eliminated through the temporary depletion of peripheral B-cells, an effect which is lasting in the long term. The only difference is the fact that this effect does not occur in a straightforward manner; it needs time: weeks to months. Yet, rituximab cannot touch plasmablasts or plasma cells, as these cells do not have CD20 receptors while they reside out of the circulation. As a result, in patients who are treated with rituximab, ANCA titers decrease gradually [27,115,116]. Nephrologists who have extensive clinical experience with these diseases are aware of the characteristics of kidney involvement, which is extremely common in ANCA-vasculitis, i.e., pauci-immune, necrotizing, and crescentic GN. Time is a significant factor here, and the glomeruli need to be rid of ANCAs as soon as possible [117]. Among other parameters, the rapidity with which eGFR declines around diagnosis is a guidepost that many clinicians would use to provide aggressive immunosuppression beyond rituximab. Even more importantly, rituximab is not a kinder or gentler immunosuppressant since results from well-designed clinical trials agree that the adverse event profile for rituximab or cyclophosphamide-based remission induction regimens is not different. The parallel profile in adverse events seem confusing given that rituximab specifically and temporarily depletes CD20-positive B-cells, while cyclophosphamide, an alkylating agent that crosslinks DNA strands, thereby decreasing DNA synthesis, casts a much larger and less specific cytotoxic effect [27,115].

Advancements in the treatment of EGPA includes the combined use of cyclophosphamide with glucocorticoids, especially for life-threatening disease, and glucocorticoids alone for patients with minor manifestations [113]. Most recently, anti-interleukin 5 therapies such as mepolizumab and benralizumab have changed the clinical practice in EGPA [118].

The efficacy of mepolizumab for EGPA was confirmed in the MIRRA study, which proved mepolizumab as a remission induction agent with a glucocorticoid-sparing option in patients with multi-relapsing or refractory disease [119]. Next, the MANDARA trial showed the non-inferiority of benralizumab compared to mepolizumab in inducing remission in EGPA [120]. Both act as interleukin-5 antagonists by reducing blood eosinophil levels, generally in the range of 60–90% of baseline depending on dose.

The therapeutic target is complement system activation. What contributes the most to high rates of serious adverse events is the exposure to high doses of glucocorticoids. This is very well understood, and therefore, a lot of work has been conducted to reverse this by reducing the exposure to glucocorticoids. To implement this “steroid sparring” strategy, the newer agents must have certain characteristics, like same efficacy as glucocorticoids, speed of action, fewer adverse events, and reduced risk of a relapse [121]. Glucocorticoids have a very broad nonselective mechanism of action on various cell types, with genomic and non-genomic effects [122].

The recent development of various drugs blocking specific steps of the complement pathways has increased. Therapies target various molecules in the complement activation cascade. Eculizumab and ravulizumab are antibodies against factor C5. Avacopan is an oral inhibitor of C5a receptor (C5aR). Vilobelimab is an antibody against factor C5a. As noted above, complement activation of the alternative pathway results in C5a production, which plays a role in the pathogenesis of ANCA-vasculitis [123]. Neutrophils express the C5aR, and binding of accumulated C5a to C5aR results in neutrophil activation. There was experimental evidence that inhibition of C5a receptor can favor the course of experimental ANCA-vasculitis [124], which consequently led to various clinical studies that tried to understand the role of avacopan in the clinical treatment of these patients.

The first published study was the CLEAR study, a phase 2 randomized three-arm trial (placebo with 60 mg of prednisone vs. 30 mg avacopan twice daily plus 20 mg of prednisone vs. avacopan without prednisone), where 67 patients with newly diagnosed or relapsing GPA or MPA were enrolled. The study showed that avacopan was effective in replacing high-dose glucocorticoids [96]. The second published study, the CLASSIC trial, was another phase 2 randomized, double-blind, placebo-controlled, three-arm trial (avacopan 30 mg plus SOC vs. avacopan 10mg plus SOC vs. SOC) which enrolled 42 patients. No significant difference in adverse events between the three arms was demonstrated, and efficacy was similar, but the renal outcomes were better with avacopan 30mg compared to avacopan 10 mg; thus, it was established that 30 mg was the right dose [125]. In a phase 3 trial, the Advocate succeeded at 2021. The trial enrolled 331 patients with new or relapsing ANCA-vasculitis (both MPA and GPA), which were treated as indicated with rituximab or cyclophosphamide. The patients were randomized to either high-dose corticosteroids or avacopan (30 mg twice daily) plus low-dose glucocorticoids for 52 weeks. The remission at week 26 and sustained remission at week 52 were the primary endpoints. Adverse events, glucocorticoid-induced toxicity, timing of response, and health-related quality of life were the secondary endpoints. Regarding the primary endpoints, avacopan showed no inferiority concerning remission at week 26, and it was superior regarding the sustained remission at week 52 (65.7% vs. 54.9%, *p* < 0.0066). The infectious adverse events were similar between the two groups, and as expected, the toxicity from glucocorticoids was significantly reduced with the use of avacopan [126]. Reviewing these data, avacopan was approved at 2021 in the United States and European community as an adjunctive treatment in adults for severe active ANCA-vasculitis (Table 1).

Inhibition of complement factor C5 with the monoclonal antibody eculizumab was found effective in three case reports. In the first one, the patient responded to therapy and achieved remission only after the addition of eculizumab to the SOC [127]. In another case, eculizumab was found effective in a patient with severe ANCA-vasculitis with the combined use of rituximab [128]. The third one was a pediatric patient with a MPO-ANCA-vasculitis recurrence post-transplant who responded to eculizumab followed by ravulizumab and achieved remission [129].

Vilobelimab, a monoclonal antibody, specifically binds and inhibits the complement fragment C5a and is currently mostly used for the treatment of COVID-19 infection [130]. The IXCHANGE trial is a phase 2, randomized, double-blind study, which evaluates whether vilobelimab is efficient as a replacement for glucocorticoids in patients with GPA or MPA. It must be specially noted that, to this date, the IXCHANGE trial has only been published in an abstract form. A total of 57 patients were enrolled in this 16-week study which consisted of three arms. The study compared vilobelimab plus reduced-dose glucocorticoids to standard-dose glucocorticoids as an add-on to SOC (rituximab or cyclophosphamide) and vilobelimab alone to standard-dose glucocorticoids in the presence of rituximab or cyclophosphamide. Remission rates were similar in all the arms of the study. Furthermore, the group of patients that were treated only with vilobelimab had a lower incidence of emergent adverse effects [131] (Table 2).

## 8. Conclusions

Recent advancements in the understanding of the pathophysiology of ANCA-vasculitis have paved the way for the development and use of newer targeted therapies, which include B-cell depletion and the complement system activation inhibition. Both alter the management of patients in the short and long term and are competent at achieving therapeutic goals, which are sustained remission with good kidney survival, decrease of relapses, and last but not least, all the above with as few adverse events as possible.

## Figures and Tables

**Table 1:** Targeting the alternative complement pathway in ANCA-vasculitis with Avacopan.

Study/Author	Design	Number of Participants	Clinical Phenotype/Organ Involvement	Complement Inhibitor/Comparator	Predefined Trial Endpoints	Results
CLEAR [96]	Phase 2, Placebo-controlled three-arm RCT	*N* = 67	GPA 49% MPA 49% Renal involvement 95%	Avacopan without GC Avacopan + low-dose GC Placebo + high-dose GC	Primary: Response at 12 weeks Secondary: Renal response at 12 weeks	81%/86%/70% Effectiveness in replacing high-dose GC
CLASSIC [125]	Phase 3, double-blind, placebo-controlled three-arm RCT	*N* = 42	GPA 69% MPA 26% Renal involvement 64%	Avacopan 10 mg + SOC Avacopan 30 mg + SOC SOC only	Primary: Incidence of AEs Response to treatment at day 85 Secondary: Infections, renal response, QoL	85%/90%/100% * Similar efficacy * AEs statistically n.s. Better renal response with 30 mg avacopan
ADVOCATE [126]	Phase 3, RCT	*N* = 331	GPA or MPA under CYC or Rituximab	Avacopan Oral GC	Primary Clinical remission at week 26 Sustained remission at week 52 Secondary GC toxicity, renal response, QoL	72.3% vs. 70.1% 65.7% vs. 54.9% Superiority for sustained remission

Abbreviations. AEs: adverse events; CYC: cyclophosphamide; GC: glucocorticoid; GPA: granulomatosis with polyangiitis; n.s.: not significant; MPA: microscopic polyangiitis; QoL: quality of life; RCT: randomized controlled trial.

**Table 2:** Targeting the alternative complement pathway in ANCA-vasculitis with Eculizumab, Ravulizumab, and Vilomelivab.

Author	Type of Publication	Clinical Phenotype	Complement Inhibitor/Comparator	Results
[127]	Case report	MPA, RPGN, AKI stage 3	Eculizumab 900 mg weekly for 1 month, 1200 mg every other week thereafter plus SOC	Almost complete renal function recovery
[128]	Case report	MPA, pulmonary–renal syndrome; RPGN, AKI stage 3	Eculizumab 900 mg weekly for 1 month, 1200 mg every other week thereafter plus RTx	Partial renal response, remission of symptoms
[129]	Case report	MPA, recurrence after pediatric kidney transplantation, AKI stage 2	Eculizumab 600 mg every week for 15 days, then every 2 weeks followed by Ravulizumab 2100 mg every 2 months	Complete renal recovery after 5 months
IXCHANGE [131]	Phase 2, double-blind 2 stages RCT, N = 57 patients involved	MPA or GPA	Vilobelimab + reduced GC dose vs. standard-dose GC plus RTx or CYC Vilobelimab alone vs. standard-dose plus RTx or CYC	Vilobelimab alone 89% remission rate Vilobelimab + red. GC 77% remission rate Standard-dose GC 96% remission rate No inferiority, lesser AEs with Vilobelimab

Abbreviations. AEs: adverse events; AKI: acute kidney injury; CYC: cyclophosphamide; GC: glucocorticoid; GPA: granulomatosis with polyangiitis; n.s.: not significant; MPA: microscopic polyangiitis; QoL: quality of life; RCT: randomized controlled trial; RPGN: rapid progressive glomerulonephritis; RTx: Rituximab; SOC: standard of care.

## Data Availability

All data is contained in this article.
